# Glucose‐Responsive and Analgesic Gel for Diabetic Subcutaneous Abscess Treatment by Simultaneously Boosting Photodynamic Therapy and Relieving Hypoxia

**DOI:** 10.1002/advs.202502830

**Published:** 2025-05-28

**Authors:** Bin Huang, Honglin An, Jianfeng Chu, Shiqi Ke, Jing Ke, Yiman Qiu, Jieping Zhang, Hanqi Zhu, Jiahui Lin, Minguang Yang, Dongliang Yang, Xuejiao Song, Weilin Liu

**Affiliations:** ^1^ Academy of Integrative Medicine Fujian Key Laboratory of Integrative Medicine on Geriatrics Fujian University of Traditional Chinese Medicine Fuzhou Fujian 350122 China; ^2^ Fujian Nanping Green Pine Chemical CO., LTD Nanping Fujian 354200 China; ^3^ The Institute of Rehabilitation Industry Fujian University of Traditional Chinese Medicine Fuzhou 350122 China; ^4^ State Key Laboratory of Flexible Electronics (LoFE) & Institute of Advanced Materials (IAM) School of Physical and Mathematical Sciences Nanjing Tech University (NanjingTech) Nanjing 211816 China

**Keywords:** borneol, diabetic abscess, hydrogel, oxygen, photodynamic therapy

## Abstract

The treatment of diabetic abscess remains highly challenging due to the complex wound healing environment, which includes bacterial infection, hypoxia, and pain, severely compromising patients' quality of life. Developing effective treatment strategies to address these multifaceted issues continues to pose significant challenges. In this study, a glucose‐triggered gel with self‐producing oxygen, photodynamic behavior, and analgesic properties is employed to address the aforementioned problems. The gel utilizes the cascade reaction of glucose oxidase and catalase to convert the excessive glucose in diabetic abscess tissue into oxygen, alleviate the hypoxia in the infected tissue, and ensure the occurrence of hypoxia photodynamic behavior to generate sufficient reactive oxygen species (ROS) to combat pathogenic bacteria. Simultaneously, borneol within the gel can not only enhance the sensitivity of bacteria to ROS by regulating the oxidative stress system but also augment the antibacterial effect of ROS. Moreover, borneol can serve as an analgesic to alleviate pain in patients. In vivo experiments have demonstrated that the combination of photodynamic and borneol therapy can eliminate the bacteria at the abscess site and enable the abscess to heal completely within 12 days. Therefore, this study established a glucose‐responsive gel for a combined therapy strategy of hypoxic improvement photodynamic therapy and borneol to facilitate wound healing in diabetic abscesses.

## Graphical abstract

The work constructs a glucose‐triggered gel for diabetic abscess treatment. The results demonstrate that the gel catalyzes glucose into oxygen, alleviates the hypoxia, and ensures sufficient ROS generation to combat pathogenic bacteria. Simultaneously, borneol within the gel can enhance the antibacterial activity of ROS and alleviate pain in patients, which offers an alternative strategy for diabetic abscess management.

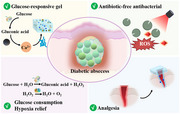

## 1 Introduction

Diabetes, a chronic metabolic disease, has a serious impact on human life and health.^[^ ^1^ ^]^ Persistent hyperglycemia can cause skin and soft tissue damage, increasing susceptibility to infections in diabetic patients.^[^ ^2^ ^]^ In 2011, Hospitalizations for diabetes‐related infections cost the U.S. healthcare system an estimated $48 billion, with costs steadily rising in correlation with increasing diabetes cases.^[^ ^3^ ^]^ Among these infections, abscesses account for over 50% of cases, with incidence rates steadily increasing.^[^ ^4^ ^]^ Globally, methicillin‐resistant Staphylococcus aureus (MRSA) is a leading cause of skin abscesses, presenting with erythema, swelling, and pain.^[^ ^4^, ^5^ ^]^ The current standard treatment involves incision and drainage with adjunct antibiotic therapy.^[^ ^6^ ^]^ While incision and drainage effectively reduce bacterial load, the procedure is invasive and painful, and antibiotic resistance often allows MRSA to persist, leading to frequent recurrence.^[^ ^7^ ^]^ Therefore, developing more effective and comfortable treatment methods is critical to improving outcomes for diabetic patients with skin abscesses.

Photodynamic antibacterial therapy as an emerging strategy has caught people's attention owing to its merits of high spatiotemporal selectivity and minor drug resistance.^[^ ^8^ ^]^ In particular, photodynamic therapy has been used in clinical applications, such as in the treatment of condylomata acuminata, actinic keratosis, acne, and so on.^[^ ^9^ ^]^ Previous evidence confirms that photodynamic therapy can efficiently eliminate drug‐resistant bacteria and their biofilm by using reactive oxygen species (ROS).^[^ ^10^ ^]^ Nevertheless, the antibacterial activity of oxygen‐dependent type‐II photodynamic therapy is significantly limited in the hypoxic diabetic wound.^[^ ^11^ ^]^

Oxygen content is not only the main parameter that influences the therapeutic effect of photodynamic therapy, but also an important parameter that determines the speed of wound healing.^[^ ^12^ ^]^ For example, oxygen is involved in the regulation of drug resistance, oxidative stress, angiogenesis, energy metabolism, and so on.^[^ ^13^ ^]^ Up to now, many materials with oxygen supply capacity (*e.g*, metal peroxides, nanozymes, perfluorocarbon, and hemoglobin) have been exploited to increase the local oxygen level.^[^ ^12b^ ^]^ Among the oxygen delivery strategies, the therapeutic platform with continuous oxygen supply ability can significantly enhance diabetic wound healing.^[^ ^14^ ^]^ To achieve this goal, some cascade catalytic nanoplatforms were developed to relieve the hypoxia by using the biochemical molecules in the lesion.^[^ ^15^ ^]^ For example, a glucose oxidase (Gox) and catalase (CAT)‐loaded rGCP nanogel developed by He et al. converts glucose (Glu) into oxygen by a cascade reaction to ensure adequate ROS generation in an anaerobic physiological environment.^[^ ^16^ ^]^ Meanwhile, in diabetic wounds, Glu can be consumed sustainably to improve oxygen deficiency, further promoting wound healing.^[^ ^17^ ^]^

Although sufficient amounts of ROS can kill cancer cells and bacteria, ROS stimulation can also induce the expression of cellular antioxidant systems, heat shock proteins, and stress‐responsive proteins to alleviate cell damage caused by ROS‐mediated antimicrobial therapy.^[^ ^18^ ^]^ To overcome intrinsic oxidative resistance, the therapeutics that can interfere with antioxidant response are generally used to boost the photodynamic therapeutic effect.^[^ ^19^ ^]^ Borneol (NB), a natural biomolecule, can be separated and purified from Chinese herbal medicine.^[^ ^20^ ^]^ Currently, NB is widely used as an additional adjuvant with extensive commercial applications across various industries, including food, cosmetics, and pharmaceuticals.^[^ ^21^ ^]^ Previous studies confirm that NB‐treated cancer cells are more sensitive to chemotherapeutics due to the imbalance of intracellular oxidative homeostasis.^[^ ^22^ ^]^ Recent studies have found that NB can also work synergistically with antibiotics to effectively kill bacteria and even drug‐resistant bacteria.^[^ ^20^, ^23^ ^]^ In addition, NB can also exert analgesic effects when combined with other drugs, thereby relieving the pain of patients during treatment.^[^ ^24^ ^]^

**Scheme 1:**
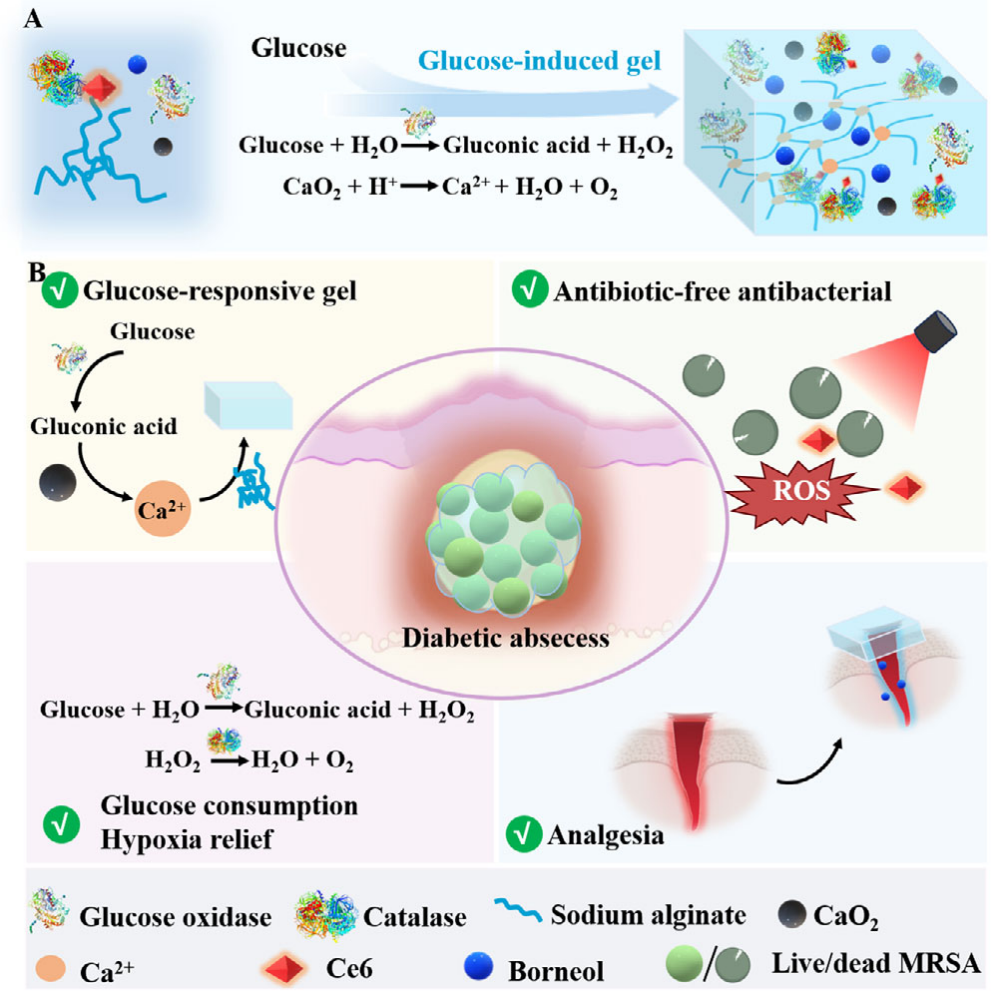
A) The preparation process of gel(CaO_2_/alg/Gox/Glu/NB/CAT‐Ce6). B) Glucose‐responsive gel for antibiotic‐free inactivation of bacteria, hypoxia relief, and analgesia in diabetic abscess wound management. The scheme was created using “BioRender”.

In this work, a Glu‐triggered gel (gel(CaO_2_/alg/Gox/Glu/NB/CAT‐Ce6)) with oxygen supply, analgesic ability, and NB‐sensitized photodynamic therapy was developed for the treatment of diabetic abscess. When the gel precursor solution (solution(CaO_2_/alg/Gox/NB/CAT‐Ce6)) is triggered by Glu, the Gox within the solution oxidizes it to form gluconic acid and hydrogen peroxide (H_2_O_2_). The resultant gluconic acid leads to the degradation of calcium peroxide (CaO_2_), and the released calcium ions can be cross‐linked with sodium alginate (alg) to form a gel. Simultaneously, the produced H_2_O_2_ can be catalyzed by CAT to generate oxygen. The generated oxygen can not only ameliorate the hypoxic environment of diabetic abscess but also promote photodynamic behavior in the hypoxic biofilm environment. Additionally, NB in the gel not only sensitizes bacteria to enhance photodynamic therapy but also alleviates the pain of patients. Therefore, once the gel is applied to diabetic abscesses, it can not only improve the oxygen depletion at the infected site to ensure the advancement of photodynamic behavior. At the same time, under 660 nm laser irradiation, NB‐enhanced photodynamic therapy can effectively eliminate drug‐resistant bacteria while relieving patients' pain, offering a novel solution strategy for the treatment of diabetic‐infected abscesses (**Scheme** **1**).

## 2 Results and Discussion

### 2.1 Preparation of CaO_2_

**Figure 1:**
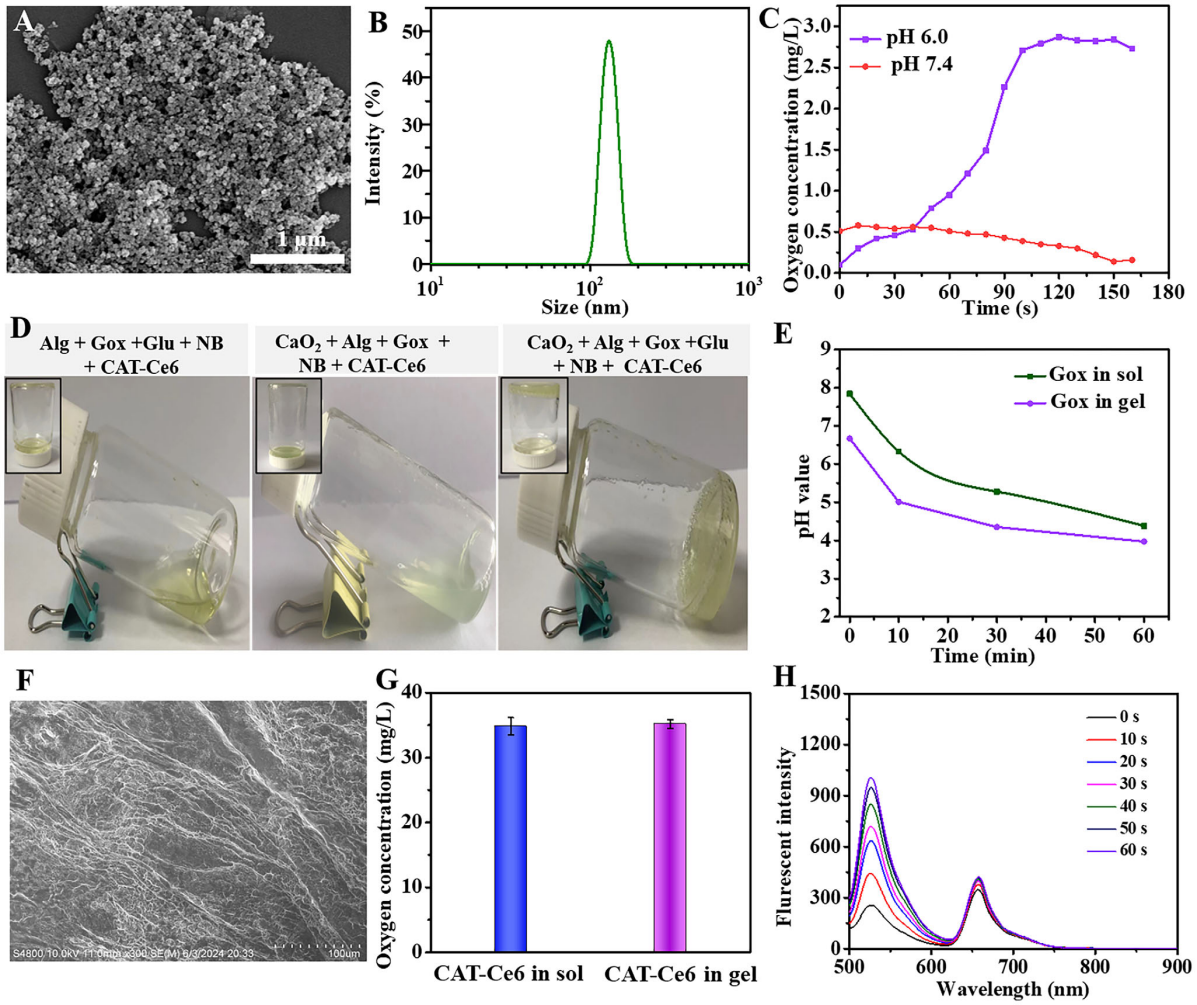
A) The SEM image and B) the diameter distribution of CaO_2_ nanoparticles. C) pH‐responsive performance of CaO_2_ nanoparticles. D) Gel photos of various precursor solutions. E) The catalytic performance of Gox in gel and solution (sol) was evaluated by monitoring the fluctuation of the pH value. F) The SEM image of gel(CaO_2_/alg/Gox/Glu/CAT‐Ce6). G) The catalytic performance of CAT in gel and solution (sol) was assessed by monitoring the generation of oxygen. Data are presented as mean ± SD, n = 3. H) The analysis of ROS generation ability of gel(CaO_2_/alg/Gox/Glu/CAT‐Ce6) by using the SOSG probe.

CaO_2_ nanoparticles were prepared according to our literature with minor modifications. Then the surface of CaO_2_ was functionalized by polyvinyl pyrrolidone (PVP) to enhance its colloidal stability. As shown in **Figure** **1**A,B, the synthesized CaO_2_ nanoparticles have a uniform diameter of ≈80 nm. The dynamic light scattering (DLS) data indicated that the hydrated particle size of the PVP‐modified CaO_2_ nanoparticles was around 130 nm. This is because in the solution state, the surface‐functionalized polymer is in a dilated state, resulting in the DLS particle size typically being higher than under dry conditions.^[^ ^25^ ^]^ Compared with traditional CaO_2_ nanoparticles, the nanoparticles can exist stably under neutral conditions and decompose to form oxygen and calcium ions under acidic conditions (Figure 1C).

### 2.2 Preparation and Properties of Glucose‐Responsive Gel

The natural product sodium alginate (alg) has been extensively researched in the field of biomedical engineering due to its excellent biosafety. The alg solution interacts with Ca^2+^ to form a Ca^2+^‐alg complex, inducing a sol‐gel transition and resulting in hydrogel formation. To fabricate an acid‐responsive antibacterial hydrogel, CaO_2_ nanoparticles were chosen as a pH‐responsive accessory and blended with the alg precursor solution. As depicted in Figure 1D, the precursor solution forms a gel only when CaO_2_, alg, Gox, and Glu are present in the precursor solution. This is because Gox can oxidize Glu to generate H₂O₂ and gluconic acid under the influence of oxygen, making the solution acidic (Figure 1E), triggering the decomposition of CaO_2_ nanoparticles, generating Ca^2+^, and thereby inducing the formation of a gel from alg solution. As shown in Figure S1A (Supporting Information), after 10 h of immersion, the gel(CaO_2_/alg/Gox/Glu/CAT‐Ce6) exhibited a swelling ratio of ≈70%, and the gel degraded after 6 days (Figure S1B, Supporting Information). For morphology characterization, the resultant gel(CaO_2_/alg/Gox/Glu/CAT‐Ce6) was freeze‐dried and exhibited wrinkles on its surface (Figure 1F). As shown in Figure 1G and Figure S2 (Supporting Information), there was no substantial alteration in the catalytic activity of the Gox enzyme before and after gelation. Additionally, to alleviate the low oxygen environment at the infection site, the CAT enzyme was incorporated into the gel, which can convert the hydrogen peroxide produced by Gox into oxygen (Figure 1G). The generation of oxygen in the later stage is not only conducive to the occurrence of photodynamic behavior but also can expedite the repair of damaged tissues. As demonstrated in Figure S3 (Supporting Information), under normoxic conditions, the addition of H₂O₂ had no significant effect on ROS generation. However, under hypoxic conditions, the ROS production of the gel(CaO_2_/alg/Gox/Glu/NB/CAT‐Ce6) was markedly suppressed due to the limited oxygen content. Notably, when H₂O₂ was introduced, CAT‐mediated decomposition of H₂O₂ generated oxygen, leading to a substantial increase in ROS production, which was nearly fourfold higher compared to the group without H₂O₂. These results confirmed that Ce6 photosensitizers in the alg‐based hydrogel can still effectively generate ROS for the removal of infectious pathogens even under anaerobic conditions (Figure 1H).

### 2.3 Optimizing of Photosensitizer and NB Concentration

**Figure 2:**
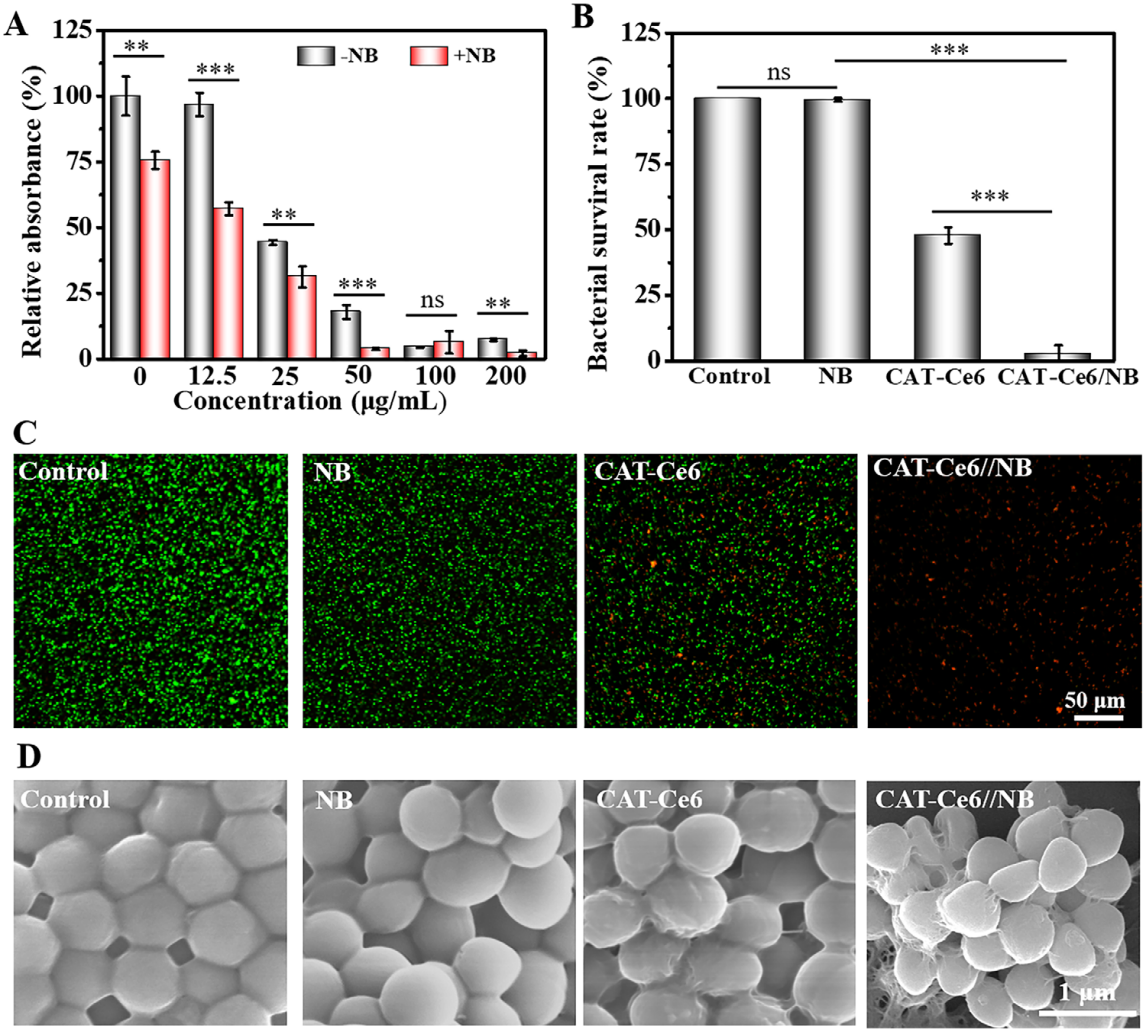
A) The combined bactericidal activity of NB and various concentrations of Ce6 against MRSA under 660 nm laser irradiation. n = 3. B) The bactericidal ability of gel(CaO_2_/alg/Glu/Gox) with different components against MRSA under 660 nm laser irradiation. n = 3. C) SYTO‐9/PI staining images and D) SEM images after MRSA treatment with different gels under 660 nm laser irradiation (Data are presented as mean ± SD. ns: no significance difference, n = 3, *P < 0.05, **p < 0.01, and ***p < 0.001).

Previous studies confirm that Ce6‐based photodynamic therapy can eliminate pathogens effectively by generating ROS to kill bacteria; meanwhile, NB can act synergistically with other therapeutics to enhance its anti‐tumor and antibacterial effects by disrupting cellular redox homeostasis.^[^ ^26^ ^]^ Thus, the synergistic effect between the photodynamic therapy and NB was investigated. As shown in Figure S4 (Supporting Information), a high concentration of NB possessed a certain antibacterial property. Approximately 25% of bacteria were inhibited upon the concentration of NB reached 1.25 mg mL^−1^. Then, the 1.25 mg mL^−1^ of NB was chosen to probe the synergistic effect of NB and photodynamic therapy. Before the investigation of the phototoxicity of CAT‐Ce6, different concentrations of CAT‐Ce6 were incubated with MRSA, and the bacterial growth was not influenced in the dark environment (Figure S5, Supporting Information). However, with the assistance of NB (1.25 mg mL^−1^) and laser irradiation, 50 µg mL^−1^ of CAT‐Ce6 should inhibit 90% of bacteria. Nevertheless, in the single treatment, to achieve a 90% inhibition effect, it needed 5 mg mL^−1^ of NB and 100 µg mL^−1^ of CAT‐Ce6 (**Figure** **2**A). To evaluate the interaction relation of NB and photodynamic therapy, the CI was determined to be 0.87, which demonstrated the synergistic antibacterial effect of photodynamic therapy and NB. Thus, during the gel preparation process, 1.25 mg mL^−1^ of NB and 50 µg mL^−1^ of Ce6 were used in further studies. To uncover the underlying mechanisms of NB synergistically enhanced photodynamic therapy, the NB‐treated MRSA was collected for RNA sequencing. 2758 genes were detected, and 867 genes exhibit significantly different expression (FDR < 0.05) upon comparison with the control group (Table S1; Figures S6 and S7, Supporting Information). Among them, the expression of 468 genes was increased and 399 genes were decreased, which confirmed that the bacterial life activities were significantly influenced after NB treatment. To uncover the interaction mechanism, differentially expressed genes (DEGs) were analyzed and classified by using the Kyoto Encyclopedia of Genes and Genomes (KEGG) database. As shown in Figure S8 (Supporting Information), the NB primarily influenced the genes involved in bacterial metabolism (e.g., energy, amino acids, lipids, nucleotides, and carbohydrates), quorum sensing, and the antibiotics synthesis pathway. Meanwhile, the DEGs' functions were classified into molecular functions (MF), cellular components (CC), and biological processes (BP) according to the Gene Ontology (GO) database (Figure S9, Supporting Information). In terms of MF, NB is mainly involved in the regulation of binding, catalytic activity, molecular function regulator, structural molecule activity, transcription regulator activity, transporter activity, and translation regulator activity. As for the CC, the genes related to the cellular anatomical entity, intracellular, and protein‐containing complex were significantly influenced. In the BP section, NB influenced the genes associated with biological regulation, cellular process, developmental process, growth, interspecies interaction between organisms, localization, metabolic process, multi‐organism process, and response to stimulus. By dissecting the genes involved in stimulus‐response, we found that the genes related to oxidative stress were changed significantly. For example, *CtsR*, a repressor of stress‐specific proteins, showed upregulated expression;^[^ ^27^ ^]^ *ScdA*, which encodes an iron‐containing protein essential for repairing ROS‐induced damage, was downregulated;^[^ ^28^ ^]^ *Prli42* knockout increases bacterial sensitivity to oxidative stress, and its expression was downregulated following NB treatment.^[^ ^29^ ^]^ Genetic deletion or transcriptional suppression of *Asp23* led to cell wall attenuation and increased oxidative stress sensitivity.^[^ ^30^ ^]^ Notably, NB treatment downregulated *Asp23* expression in *S. aureus*. And the results were also confirmed by quantitative polymerase chain reaction (Table S2; Figure S10, Supporting Information). These results indicate NB‐enhanced photodynamic therapeutic effect by inhibiting the expression of antioxidant genes and interfering with bacterial adaptability.

### 2.4 The Bactericidal Activity of the Gel(CaO_2_/alg/Gox/Glu/NB/CAT‐Ce6)

The bactericidal performance of the gels against MRSA was assessed. In the absence of laser irradiation, all gels showed no apparent antibacterial activity (Figure S11, Supporting Information). Under laser irradiation, the control group of gel(CaO_2_/alg/Gox/Glu) and the treatment group of gel(CaO_2_/alg/Gox/Glu/NB) did not exhibit bactericidal properties toward MRSA. However, in the gel(CaO_2_/alg/Gox/Glu/CAT‐Ce6), the bactericidal rate against MRSA is approximately 53.3%. In contrast, the gel(CaO_2_/alg/Gox/Glu/NB/CAT‐Ce6) exhibits excellent bactericidal activity, removing ≈97.5% of the bacteria (Figure 2B). For Gram‐negative *E. coli*, the gel(CaO_2_/alg/Gox/Glu/NB/CAT‐Ce6) showed greater antibacterial efficacy, which may be attributed to the thinner peptidoglycan layer of Gram‐negative bacteria compared to Gram‐positive bacteria (Figure S12, Supporting Information). In addition, the bactericidal activity of gels was confirmed by the SYTO 9/PI Live/Dead Stain Kit. As presented in Figure 2C, in both the control and gel(CaO_2_/alg/Gox/Glu/NB) treatment groups, most of the bacteria emit green fluorescence (SYTO‐9), and almost none of the bacteria are stained with red fluorescent dye (PI), indicating that the treated bacteria above have a complete membrane structure. On the contrary, in the gel with CAT‐Ce6 treatment groups, light can activate the Ce6 photosensitizer to generate ROS and kill bacteria, so some of them are stained red fluorescence by PI; especially in the gel‐treated group containing both CAT‐Ce6 and NB, only red fluorescence can be seen throughout the picture, indicating that in this treatment group, the bacterial membrane was destroyed by ROS, leading to bacterial death. The SEM images show that the bacteria in the gel(CaO_2_/alg/Gox/Glu) and gel(CaO_2_/alg/Gox/Glu/NB)‐treated groups feature an intact bacterial membrane and a rounded, sleek surface (Figure 2D). Compared with the control group, the bacterial surface of the gel(CaO_2_/alg/Gox/Glu/CAT‐Ce6) group exhibited obvious wrinkles and shape deformation, especially in the gel (CaO_2_/alg//Gox/Glu/NB/CAT‐Ce6) group. This suggests that the combination of photodynamic therapy and NB can enhance the destructive effect on the bacterial membrane.

**Figure 3:**
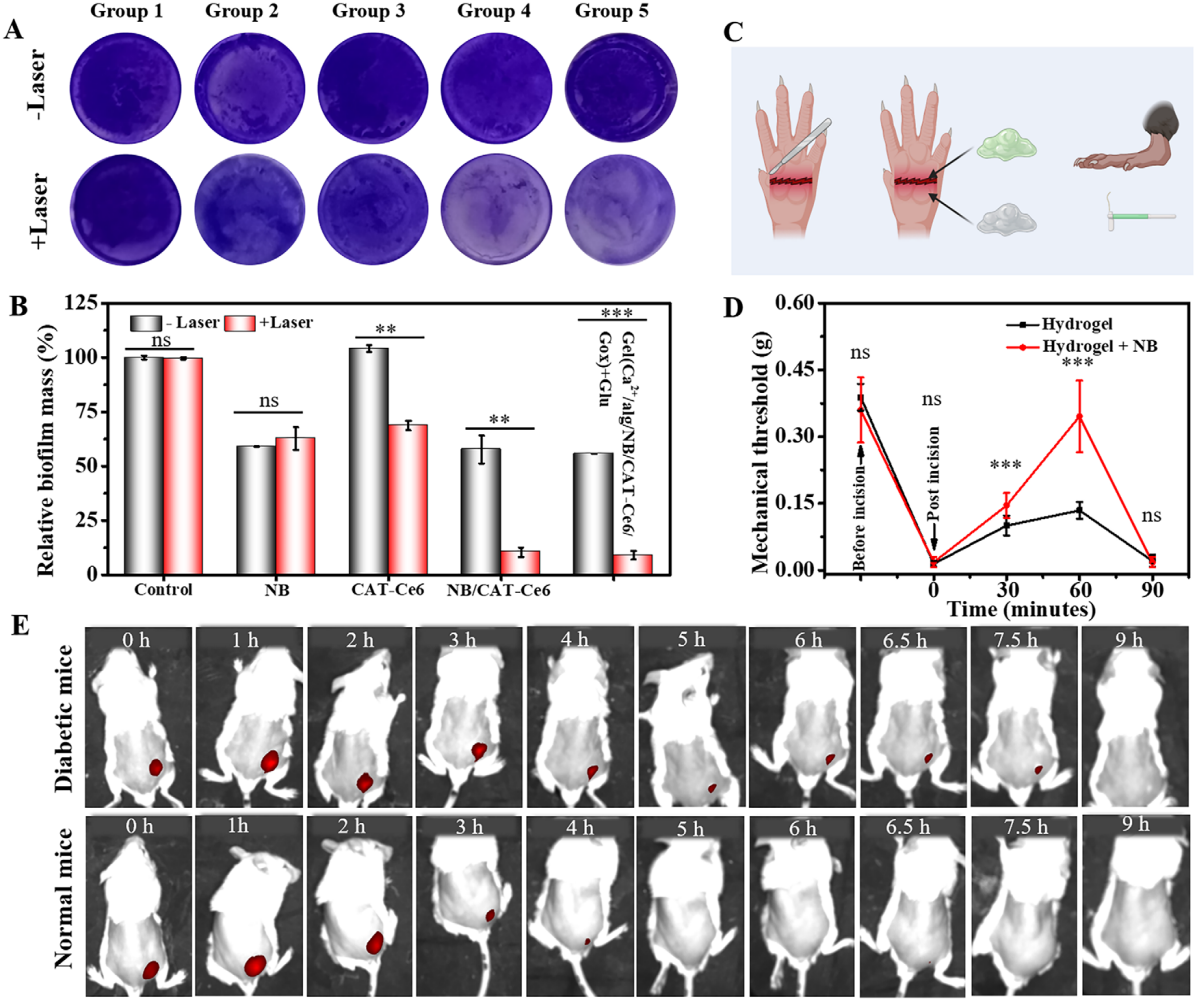
A) Crystal violet‐stained images and B) corresponding analysis of MRSA biofilms after receiving different treatments. Groups 1–5 represent gel(CaO_2_/alg/Gox/Glu), gel(CaO_2_/alg/Gox/Glu/NB), gel(CaO_2_/alg/Gox/Glu/CAT‐Ce6), gel(CaO_2_/alg/Gox/Glu/NB/CAT‐Ce6), gel(Ca^2+^/alg/Gox/NB/CAT‐Ce6) + Glu, respectively. n = 3. C) The schematic image of analgesic analysis. Created with “BioRender”. D) Analgesicr performance of gel(CaO_2_/alg/Gox/Glu/NB/CAT‐Ce6) group in incision wound. n = 13. E) In vivo fluorescence images of mice after subcutaneous injection of (CaO_2_/alg/Gox/NB/CAT‐Ce6) precursor solution (Data are presented as mean ± SD. ns: no significance difference, n = 3, *P < 0.05, **p < 0.01, and ***p < 0.001).

Bacteria can accumulate and multiply at the interface, secreting extracellular matrix to protect themselves and thereby forming bacterial biofilms. The existence of bacterial biofilms is one of the main causes of the low efficacy of conventional antibiotics. Hence, assessing the antibacterial biofilm performance of gels is of utmost significance for the treatment of biofilm‐related infectious diseases. Here, we evaluated the antibacterial biofilm activity of the gel through the crystal violet staining method. In the absence of light, the gel(CaO_2_/alg/Gox/Glu/NB) releases NB, which eliminates ≈44% of the bacterial biofilm (Figures S13 and S14, Supporting Information). Under light, the gel(CaO_2_/alg/Gox/Glu/CAT‐Ce6) removed 42% of the biofilm. In the gel(CaO_2_/alg/Gox/Glu/NB/CAT‐Ce6) group, ≈ 90% of the bacterial biofilm was removed (**Figure** **3**A,B). Against Gram‐negative *E. coli*, the gel(CaO_2_/alg/Gox/Glu/NB/CAT‐Ce6) also demonstrates biofilm‐clearing activity (Figure S15, Supporting Information). Additionally, to verify that the gel can utilize ambient Glu, gel(Ca^2+^/alg/Gox/NB/CAT‐Ce6) + Glu was compared with gel(CaO_2_/alg/Gox/Glu/NB/CAT‐Ce6), and the presence of Glu in the gel or the environment had no notable effect on its antibiofilm activity.

### 2.5 In Vivo Evaluation of Analgesic Efficacy and Gel Formation

During the treatment of subcutaneous abscesses in clinical practice, analgesic drugs are often required to alleviate the pain of patients.^[^ ^31^ ^]^ The analgesic activity of traditional Chinese medicine NB has been reported.^[^ ^24^ ^]^ Thus, we test whether the gel containing NB can relieve wound pain. As demonstrated in the von Frey test (Figure 3C,D), the gel(CaO_2_/alg/Gox/Glu/NB/CAT‐Ce6) showed the effect of alleviating mechanical allodynia. After a small incision was made on the paw pad with a knife, the gel was applied. Compared with the control group of gel(CaO_2_/alg/Gox/Glu/ CAT‐Ce6), the mechanical thresholds were significantly increased in the gel(CaO_2_/alg/Gox/Glu/NB/CAT‐Ce6)‐treated group within 30 to 90 min, but then decreased over time. These results suggest that the gel(CaO_2_/alg/Gox/Glu/NB/CAT‐Ce6) can relieve mechanical pain.

To demonstrate that the gel precursor solution can gel in vivo, the CaO_2_/alg/Gox/NB/CAT‐Ce6 precursor solution was subcutaneously injected into diabetic mice. Two hours after the injection, the subcutaneous tissue was collected. As shown in Figure S16 (Supporting Information), the subcutaneous tissue was covered with a gel layer, indicating that the gel system could also be activated by hyperglycemia in the body and then transformed into gel. Additionally, Ce6 fluorescence was employed to observe the retention of the generated hydrogel in vivo. As shown in Figure 3E, compared with normal mice, the Ce6 fluorescence in diabetic mice had a longer retention time in the body. This might be because the high glucose environment in diabetic mice creates a relatively acidic environment, which is more conducive to calcium ion release and gel formation.

### 2.6 The In Vivo Therapeutic Performance

**Figure 4:**
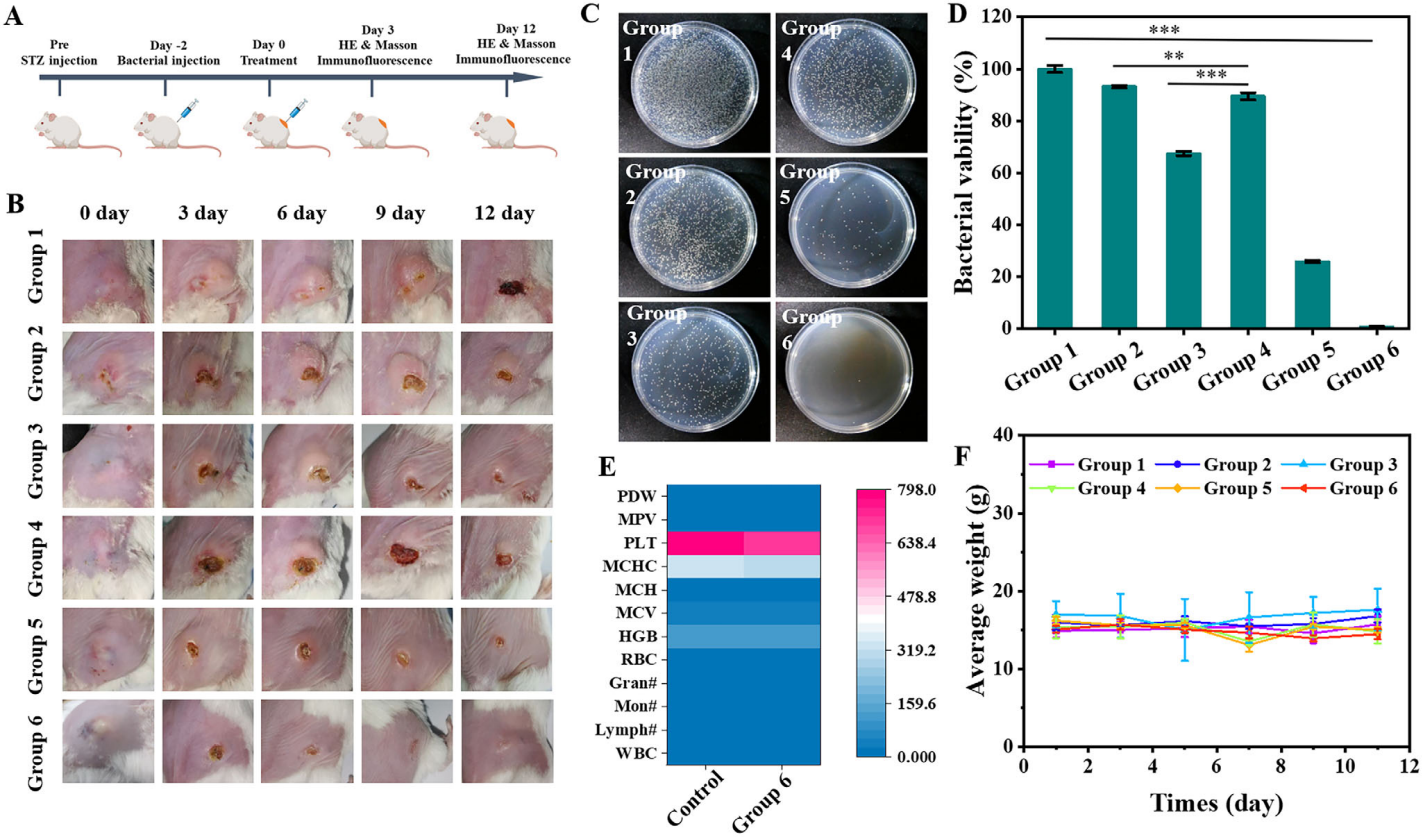
A) Schematic illustration of the management of diabetic subcutaneous abscess. Created with “BioRender”. B) Abscess images after different treatments. MRSA survival in the abscess after different treatments, C) Plate photographs and D) corresponding colonies statistical analysis. E) Blood routine test on day 12, control: normal mice. F) Body weight fluctuation during treatment. Group 1–6: no treatment (group 1), CaO_2_ + alg precursor solution (group 2), CaO_2_/alg/Gox precursor solution (group 3), CaO_2_/alg/CAT‐Ce6 precursor solution (group 4), CaO_2_/alg/Gox/CAT‐Ce6 precursor solution (group 5), and CaO_2_/alg/Gox/CAT‐Ce6/NB precursor solution (group 6) (Data are presented as mean ± SD. ns: no significance difference, n = 5, *P < 0.05, **p < 0.01, and ***p < 0.001).

To construct the diabetic mice model, streptozotocin was subcutaneously administered, and the changes in blood glucose concentration were monitored in real‐time using a glucometer. When the blood glucose concentration exceeds 15 mmol L^−1^, it is considered that the mouse diabetes model has been successfully established. Subsequently, *S. aureus* was injected subcutaneously to construct a subcutaneous abscess model. Then, the gel precursor solution, including different components, was injected in situ and induced to gel under the hyperglycemic condition. After treatment, the changes in the number of bacteria at the infection site and tissue recovery were detected to evaluate the therapeutic performance of the hydrogel. The specific flowchart is shown in **Figure** **4**A. As depicted in Figure 4B, the abscesses in mice of the control group increased significantly, and part of the apostematic skin ulcerated and formed crusts. As shown in Figure 4B and Figure S17 (Supporting Information), in the CaO_2_ + NB + CAT‐Ce6 + Gox (gel(CaO_2_/alg/Gox/Glu/NB/CAT‐Ce6), group 6) treatment group, the volume of mouse abscesses decreased significantly, and the abscess disappeared by day 9. After 12 days of treatment, the amounts of residual bacteria in the CaO_2_ + alg + Gox, CaO_2_ + alg + CAT‐Ce6, and CaO_2_ + alg + CAT‐Ce6 + Gox groups (i.e., groups 3, 4, and 5) were approximately 68%, 90%, and 25%, respectively, while no bacteria were detected in the CaO_2_ + Alg + NB + CAT‐Ce6 + Gox group (i.e., group 6) (Figure 4C,D). These results suggest that the incorporation of Gox in the hydrogel enables it to convert the excessive glucose in the patient's body to generate H_2_O_2_, which produces oxygen under the action of CAT, enhancing the photodynamic antibacterial effect under anaerobic conditions. Simultaneously, the addition of NB can not only alleviate wound pain but also enhance the sensitivity of bacteria to photodynamic therapy, thereby effectively eliminating bacteria in the abscess and accelerating the resolution of the abscess. At the same time, during the treatment process, the in vivo toxicity of the gel(CaO_2_/alg/Gox/Glu/NB/CAT‐Ce6) was also preliminarily explored. As depicted in Figure 4E,F, the blood routine analysis result of the mice exhibited no obvious abnormalities, and the weight of the mice remained stable without significant fluctuations during the treatment. In addition, the histochemical analysis revealed no significant pathological alterations in the major organs of the mice (Figure S18, Supporting Information). The above outcomes indicated that gel(CaO_2_/alg/Gox/Glu/NB/CAT‐Ce6) presented no obvious toxicity during the treatment, suggesting that this hydrogel holds a promising application prospect in biomedicine.

### 2.7 Histological Examination

**Figure 5:**
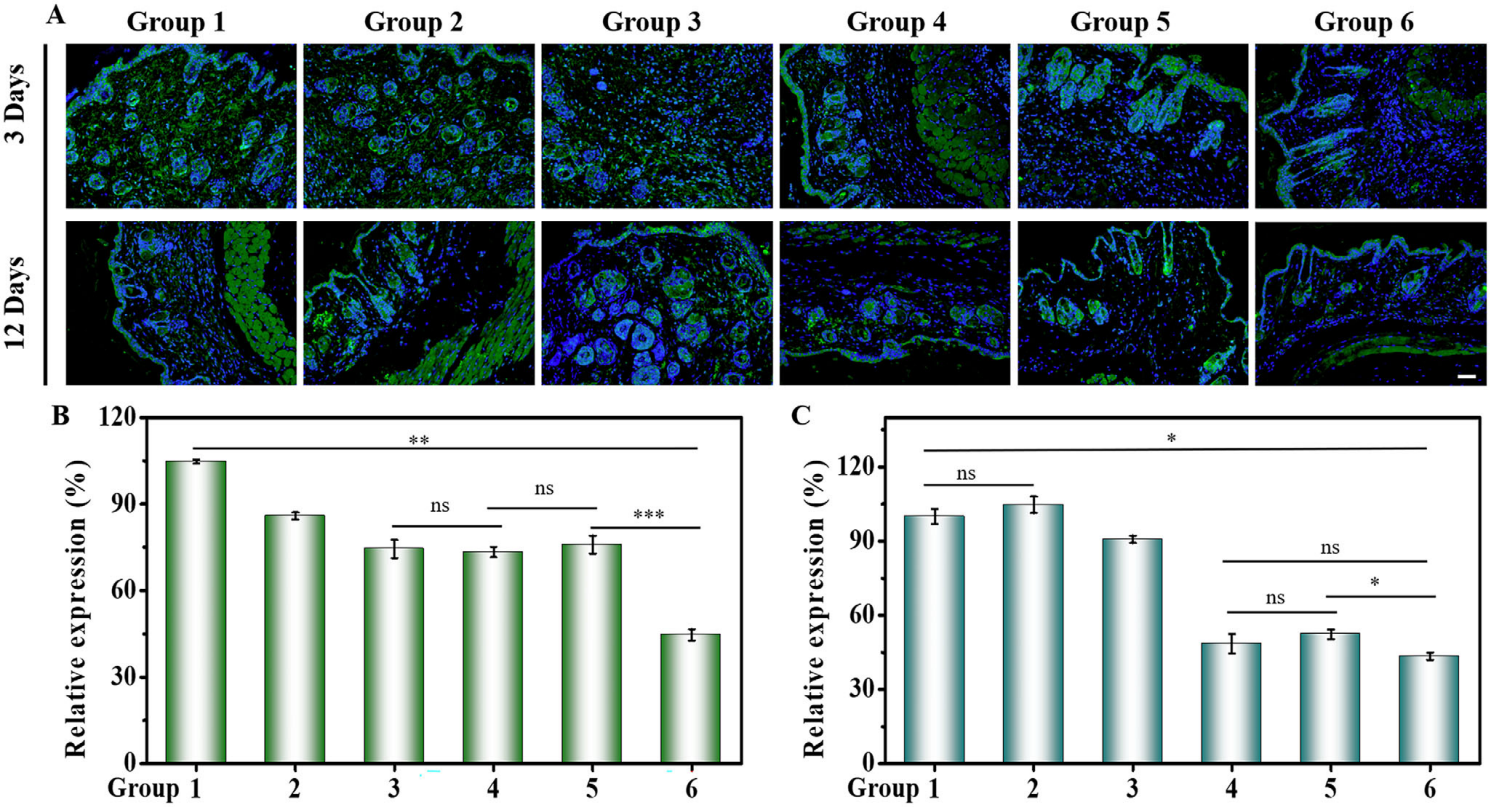
A) HIF‐1α fluorescent (green) staining images of infected tissues on days 3 and 12 after different treatments (scale bar = 50 µm) and corresponding colonies statistical analysis (B, day 3; C, day 12). Group 1–6: no treatment (group 1), CaO_2_/alg precursor solution (group 2), CaO_2_/alg/Gox precursor solution (group 3), CaO_2_/alg/CAT‐Ce6 precursor solution (group 4), CaO_2_/alg/Gox/CAT‐Ce6 precursor solution (group 5), and CaO_2_/alg/Gox/CAT‐Ce6/NB precursor solution (group 6) (Data are presented as mean ± SD. ns: no significance difference, n = 3, *P < 0.05, **p < 0.01, and ***p < 0.001).

**Figure 6:**
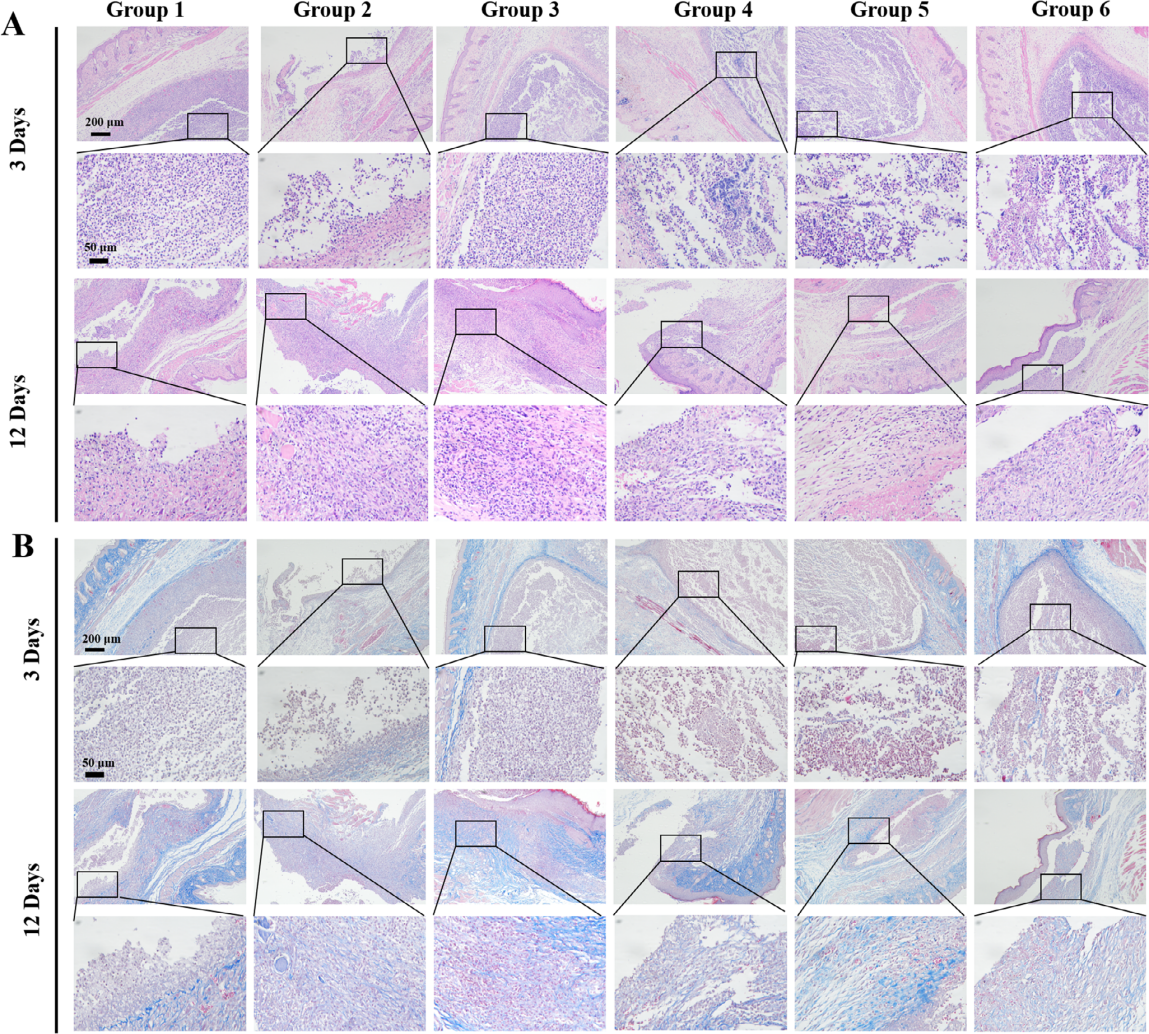
A) H&E and B) Masson trichrome staining images of infected tissues at days 3 and 12. Group 1–6: no treatment (group 1), CaO_2_/alg precursor solution (group 2), CaO_2_/alg/Gox precursor solution (group 3), CaO_2_/alg/CAT‐Ce6 precursor solution (group 4), CaO_2_/alg/Gox/CAT‐Ce6 precursor solution (group 5), and CaO_2_/alg/Gox/CAT‐Ce6/NB precursor solution (group 6).

When tissues are exposed to low oxygen, the expression of hypoxia‐inducible factor (HIF)‐1α, which is associated with oxygen regulation, is increased.^[^ ^32^ ^]^ Therefore, the fluctuation of oxygen levels in infected tissues can be indirectly investigated by measuring the change in HIF‐1α content. As shown in **Figure** **5**A, the higher expression of HIF‐1α in the control group indicated that the abscess tissue was in a relatively hypoxic environment. However, in the treatment group containing CaO_2_/alg/Gox and CaO_2_/alg/CAT‐Ce6 precursor solution (i.e., groups 3 and 4), the green fluorescence was significantly reduced, indicating that the hydrogel could produce oxygen and improve tissue hypoxia (Figure 5B,C). Oxygen concentration is significant for repairing damaged areas, and studies have shown that high oxygen concentrations can accelerate tissue healing. H&E (hematoxylin‐eosin) staining analysis was performed on infected tissues to evaluate tissue healing.^[^ ^33^ ^]^ After 3 days of treatment, all groups had a significant inflammatory response. With the extension of treatment time (6 days), there were almost no excessive inflammatory cells in the subcutaneous abscess tissue of gel(CaO_2_/alg/Gox/Glu/CAT‐Ce6) and gel(CaO_2_/alg/Gox/Glu/NB/CAT‐Ce6) treatment groups (i.e., group 5 and 6), indicating that the hydrogel can significantly accelerate the healing of infected tissue (**Figure** **6**A). In addition, collagen, as an important component of the skin, plays an important role in tissue remodeling. The process of tissue remodeling can be understood through qualitative or quantitative analysis of collagen regeneration in the damaged part.^[^ ^34^ ^]^ No significant collagen deposition can be observed at the site of the abscess on day 3. On day 12, as shown in Figure 6B, collagen deposition in the gel(CaO_2_/alg/Gox/Glu/CAT‐Ce6) and gel(CaO_2_/alg/Gox/Glu/NB/CAT‐Ce6) groups (i.e., groups 5 and 6) increased significantly compared with the control group. These results indicated that this hydrogel could eliminate the bacteria at the infection site and utilize the high concentration of glucose at the infection site to generate oxygen to alleviate the internal oxygen deficiency, thereby accelerating the healing of the damaged tissue.

## 3 Conclusion

In summary, this study synthesized a Glu‐responsive gel loaded with Gox, CAT, Ce6, and NB to eradicate traumatic infectious drug‐resistant pathogens and promote the healing of refractory abscess. After administering gel precursor solution, hyperglycemia in diabetic abscess wounds can trigger the transformation of the pre‐gel solution into a gel. By employing the cascade reaction of Gox and CAT enzymes, in situ oxygen supply to abscess sites was accomplished, which not only enhanced the occurrence of photodynamic behavior in the hypoxic infection environment but also facilitated the wound healing of abscesses. Meanwhile, NB in the gel increased the sensitivity of bacteria to ROS by regulating the oxidative stress system and alleviating the pain of patients' wounds. In vivo experiments demonstrated that the combination of photodynamic antibacterial and traditional Chinese medicine NB with the Glu‐responsive gel platform could improve hypoxia, achieve local bacterial clearance, and relieve wound pain. This study can not only enhance the treatment of diabetic wound infection but also provide a reference for the subsequent development of biomedical materials with analgesic effects.

## 4 Experimental Section

### Preparation of CaO_2_ NPs

Four milliliters of CaCl_2_ ethanol solution (0.1 g mL^−1^) and 90 mL of PEG‐200 were fully mixed in a conical flask. Then, 1.1 mL of ammonia and 2 mL of H_2_O_2_ (30%) solution were added slowly into the above solution. The resulting solution was stirred until it was clarified. Subsequently, 8 mL of NaOH solution (1 m) was added slowly to obtain a white and turbid liquid. Finally, the white product (CaO_2_ NPs) was harvested after being washed with deionized water and ethanol several times. In addition, to keep CaO_2_ NPs for a long time, the final sample was vacuum‐dried and stored in an anhydrous environment.

### Synthesis of CAT‐Ce6

To synthesize Ce6‐modified catalase (CAT‐Ce6), the carboxyl functional groups of Ce6 were activated in dimethylsulfoxide solution after being interacted with 1‐(3‐Dimethylaminopropyl)‐3‐ethylcarbodiimide hydrochloride and 1‐Hydroxy‐2,5‐pyrrolidinedione. Then, the pretreated Ce6 solution was added to the CAT solution and shaken to ensure adequate reaction of the amino groups of CAT and carboxyl groups of Ce6. After reaction for 12 h, the reaction liquid was dialyzed (molecular weight cut‐off, 14 kDa) for purifying CAT‐Ce6. For further convenient use, the synthesized CAT‐Ce6 was kept in a low‐temperature environment (4 °C) without light.

### Preparation of Gel(CaO_2_/alg/Gox/Glu/CAT‐Ce6)

To fabricate the gel(CaO_2_/alg/Gox/Glu/CAT‐Ce6), CaO_2_ NPs were added into the alg/Gox/NB/CAT‐Ce6 precursor solutions, which included 100 µg mL^−1^ CAT‐Ce6, 5 mg mL^−1^ alg, 15 mM Glu and 1.25 mg mL^−1^ NB. After adding CaO_2_ NPs, the precursor solution would gel in a few minutes. In subsequent experiments, gel(CaO_2_/alg/Gox/Glu/CAT‐Ce6) was used immediately after it was made.

### Evaluation of NB‐Sensitized Photodynamic Antibacterial Effect

To evaluate the bactericidal activity of NB and CAT‐Ce6, MRSA was incubated with various concentrations and irradiated with a 660 nm laser for 30 min according to the experimental requirement. After incubation at 37 °C for 12 h, the bacterial density was measured by using turbidimetric methods. To evaluate the NB‐enhanced photodynamic antibacterial therapy, MRSA was incubated with NB (1 mg mL^−1^) and different concentrations of CAT‐Ce6 for 6 h, then the samples were irradiated with a 660 nm laser (0.3 W cm^−2^) for 30 min and incubated for another 10 h before bacterial density assay.

To determine the synergistic effect between the NB and photodynamic antibacterial therapy (PDAT), the combination index (CI) was evaluated according to the following Equation (1):

(1) CI=CPDAT/CfPDAT+CCT/CfCT+CPDATCCT/CfPDATCfCT

In this formula, CfPDAT refers to the concentration of PDAT to achieve 90% bactericidal effect alone and CPDAT refers to the concentration of PDAT to achieve 90% bactericidal effect in combination with PDAT‐chemotherapy (CT), while CfCT refers to the concentration of CT to achieve 90% bactericidal effect alone and CCT refers to the concentration of CT to achieve 90% bactericidal effect in combination with PDAT‐CT. The CI value less than 1 indicates synergism, while the CI value greater than 1 indicates antagonism. To reveal the potential mechanism of NB‐enhanced photodynamic therapy, RNA transcriptome analysis was performed. After MRSA was treated with NB, the bacterial cell was collected for total RNA extraction by using the *Staphylococcus aureus* RNA extraction kit. The quality of extracted RNA was evaluated by using an Agilent Technologies 2100 Bioanalyzer. Subsequently, the reverse‐transcribed cDNA was transferred to Shanghai Biozeron Biothchnology Co., Ltd (Shanghai, China) for RNA sequencing analysis.

### Singlet Oxygen Detection

A commercial singlet oxygen sensor green (SOSG) was used as a fluorescent probe to analyze the singlet oxygen generation activity. Gel(CaO_2_/alg/Gox/Glu/CAT‐Ce6) was displaced in a quartz cuvette, which was preadded 2 mL of deionized water and the SOSG probe (50 µm), then the oxygen in the sample was removed by using nitrogen before receiving laser irradiation. After the sample was irradiated with a 660 nm laser, the fluorescent spectra were collected by using a fluorescence spectrophotometer.

### Evaluation of Oxygen Production Performance

Gel(CaO_2_/alg/Gox/Glu/CAT‐Ce6) was immersed in a solution containing glucose. Then, a dissolved oxygen meter was used to monitor the O_2_ concentration variation in the water.

### NB Release Behavior

NB detection was performed using a vanillin probe as previously described.^[^ ^26^ ^]^ 4.5 mL of concentrated sulfuric acid that preadded 10 mg mL^−1^ vanillin was rapidly added into 0.5 mL of the leaching solution of Gel(CaO_2_/alg/Gox/Glu/CAT‐Ce6). After mixing thoroughly, the samples were kept at 30 °C for 10 min before the absorbance value of the samples was measured by using a UV‐vis spectrophotometer.

### Antibacterial and Antibiofilm Assay

To evaluate the bactericidal properties of the gel, the samples were immersed in the MRSA bacterial solution for 6 h and subsequently irradiated with 660 nm laser (0.3 W cm^−2^) for 30 min, then the bacteria were harvested for live/dead fluorescent assay by using SYTO9/PI dual stained kit. For bacterial morphology assay, the treated bacteria were dehydrated using different concentrations of ethanol before being dropped on a silicon wafer for SEM imaging.

For the antibiofilm assay, MRSA was cultured in tryptic soy broth (TSB) medium for 48 h to harvest the mature bacterial biofilm. Then the biofilms were preincubated with the gels containing different components (NB, CAT‐Ce6, NB + CAT‐Ce6, the concentration of NB and CAT‐Ce6 were 1.25 mg mL^−1^ and 100 µg mL^−1^, respectively) for 12 h before receiving 660 nm laser irradiation (30 min). To investigate the antibiofilm activity, the biofilm biomass was quantified by using the crystal violet staining method.

### Pain Relief Assay

The animal experiments were implemented under the supervision and permission of the Fujian University of Traditional Chinese Medicine (Approval no. 2024019). Eight‐week‐old male C57/BL mice were anesthetized with sevoflurane, and an incision (3.5 mm × 1 mm) was created on the right paw pad using a scalpel. Then, the formulated gels were applied to the wound. Mechanical allodynia of mice was measured by von Frey test before incision and after awake. The von Frey test was modified according to the previous approach,^[^ ^35^ ^]^ rapid retraction or flinching of the paw was regarded as a positive control, and the amount of force required to elicit a positive response in at least 60% of the trials was documented as the mechanical threshold.

### MRSA‐Infected Diabetic Abscess Treatment

The animal experiments were approved by the Institutional Animal Care and Use Committee of Huazhong University of Science and Technology (Approval no. [2024]S4367). The newly bought mice were raised for a week to acclimate to the new environment. Then, a diabetic mouse model was constructed by injecting 60 mg kg^−1^ streptozotocin into the abdominal cavity for 5 consecutive days. During streptozotocin treatment, the mice's glucose fluctuation was monitored by a commercial glucose meter. Once the blood glucose level was higher than 16.7 mm, it indicated that the diabetes model had been created successfully.

To construct MRSA‐infected abscesses, 100 µL of MRSA bacterial suspension (10^8^ CFU mL^−1^) was injected subcutaneously into the back of anesthetized mice. One day after infection, the mice were treated with PBS, CaO_2_/alg, CaO_2_/alg/Gox, CaO_2_/alg/CAT‐Ce6, CaO_2_/alg/Gox/CAT‐Ce6, and CaO_2_/alg/Gox/CAT‐Ce6/NB precursor solution, respectively. After hydrogel administration and laser treatment, the abscess area changes were photographed and quantified using ImageJ software. Meanwhile, the weight change of the mice was checked every day. To assess the antibacterial capacity of Gel(CaO_2_/alg/Gox/Glu/CAT‐Ce6), after being treated for 3 days, the infected tissues from different groups were harvested for tissue bacterial count by using colony counting methods.

### Histologic Analysis

To investigate the effect of gel(CaO_2_/alg/Gox/Glu/NB/CAT‐Ce6) on relieving hypoxia, the diabetic infected tissues were sectioned and sliced for HIF‐1α and VEGF immunofluorescent staining after receiving treatment for 3 days. To explore the wound healing profile, the infected tissues were sliced for Masson and H&E staining. To evaluate the potential toxicity of gel(CaO_2_/alg/Gox/Glu/NB/CAT‐Ce6), sections of major organs were used for H&E staining.

### Statistical Analysis

All experimental data were visualized, analyzed, and processed in the Origin 8.5 software. One‐way ANOVA and Tukey tests were used for significance analysis (Tukey) was used for significance analysis. All experiments should be performed in at least three independent replicates. Data are presented as mean ± SD.

## Conflict of Interest

The authors declare no conflict of interest.

## Author Contributions

B.H., H.A., and J.C. contributed equally to this work. B.H. and D.Y. performed conceptualization. J.K., D.Y., and X.S. performed data curation. H.A., Y.Q., J.Z., and M.Y. performed formal analysis. B.H. performed Funding acquisition. H.A., Y.Q., H.Z., and J.L. performed investigation. D.Y. and B.H. performed methodology. W.L. performed project administration. J.C., S.K., and J.K. performed resources. S.K., J.K., and J.C. performed Supervision. H.A., Y.Q., S.K., J.K., and H.Z. performed validation. H.A. and J.C. performed visualization. B.H. wrote‐original draft. B.H., D.Y., and W.L. wrote, reviewed, and edited.

## Supporting information



Supporting Information

## Data Availability

The data that support the findings of this study are available from the corresponding author upon reasonable request.
