# Supplementary material for: Glucose‐Responsive and Analgesic Gel for Diabetic Subcutaneous Abscess Treatment by Simultaneously Boosting Photodynamic Therapy and Relieving Hypoxia
Source: Adv Sci (Weinh). 2025 May 28;12(31):e02830. doi: 10.1002/advs.202502830 (PMC12376599; doi:10.1002/advs.202502830)
Supplement: Supplementary file 1 — Supporting Information [file ADVS-12-e02830-s001.docx]

Supporting Information

Glucose-Responsive and Analgesic Gel for Diabetic Subcutaneous Abscess Treatment by Simultaneously Boosting Photodynamic Therapy and Relieving Hypoxia

Bin Huang^#^, Honglin An^#^, Jianfeng Chu^#^, Shiqi Ke, Jing Ke, Yiman Qiu, Jieping Zhang, Hanqi Zhu, Jiahui Lin, Minguang Yang, Dongliang Yang,* Xuejiao Song,* and Weilin Liu*


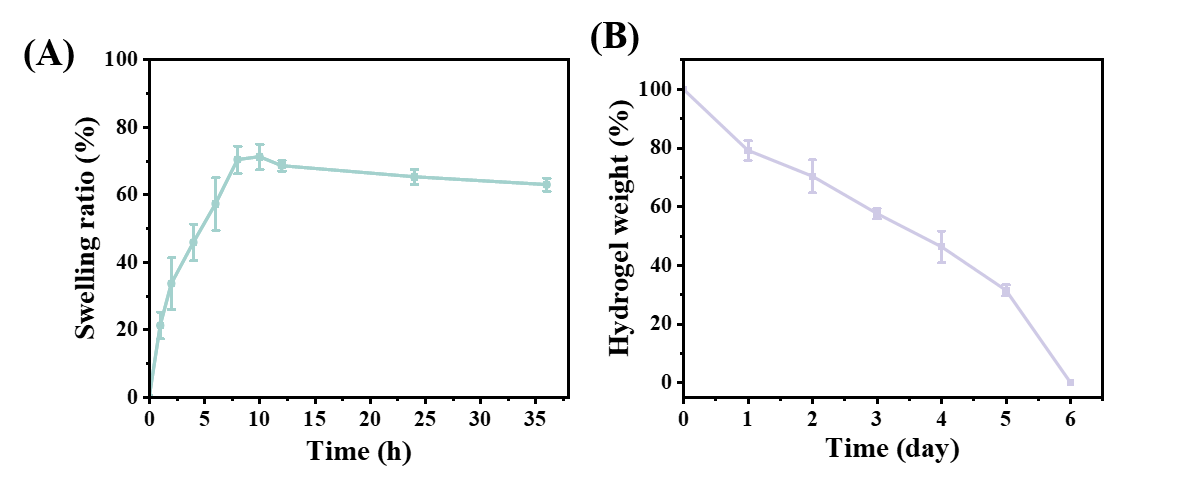


**Figure S1**. The characterization of the gel. (A) The swelling ratio of the gel. (B) The degradation ratio of the gel (Data are presented as mean ± SD, n=3).


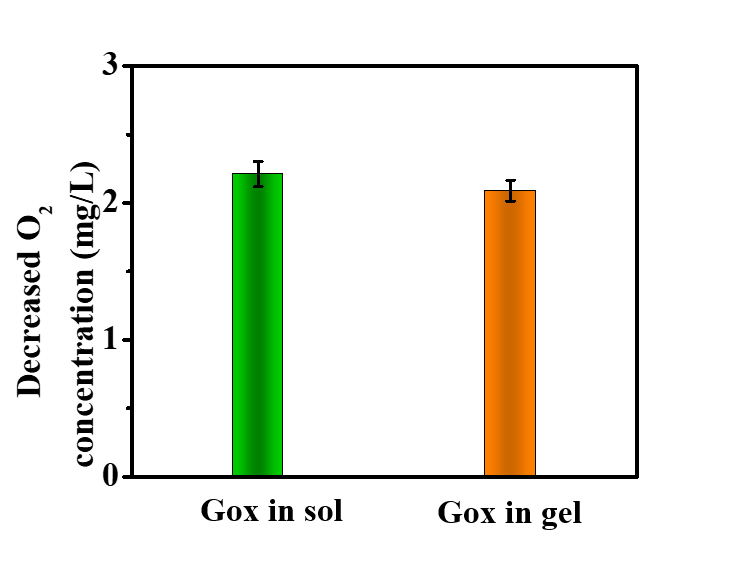


**Figure S2**. The change in oxygen concentration in gel and solution during the catalysis of glucose by Gox (Data are presented as mean ± SD, n=3).


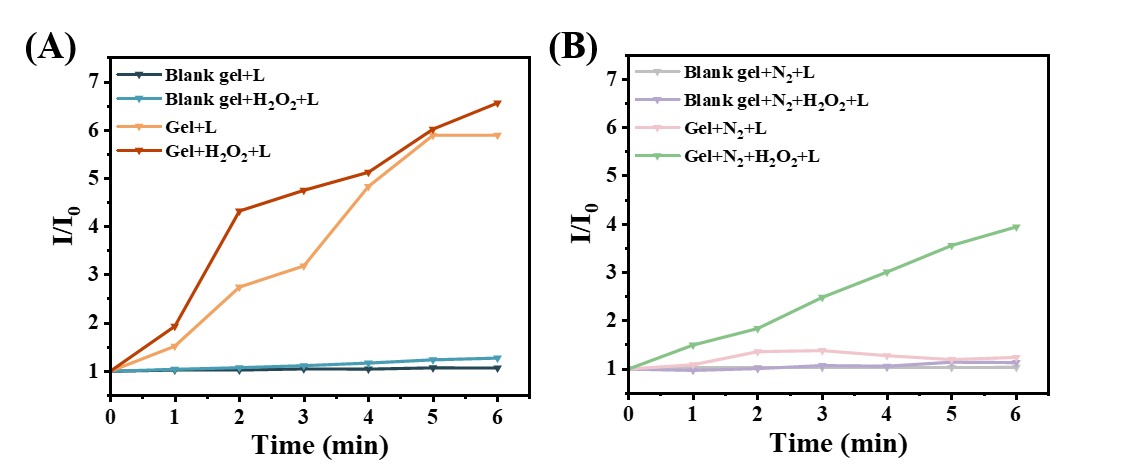


**Figure S3.** The ROS generation of the hydrogel under different conditions. (a) The ROS generation of blank hydrogel and gel(CaO_2_/alg/Gox/Glu/NB/CAT-Ce6) under normoxic conditions with or without H_2_O_2_ addition. (b) The ROS generation of blank hydrogel and gel under hypoxia conditions with or without H_2_O_2_ addition.


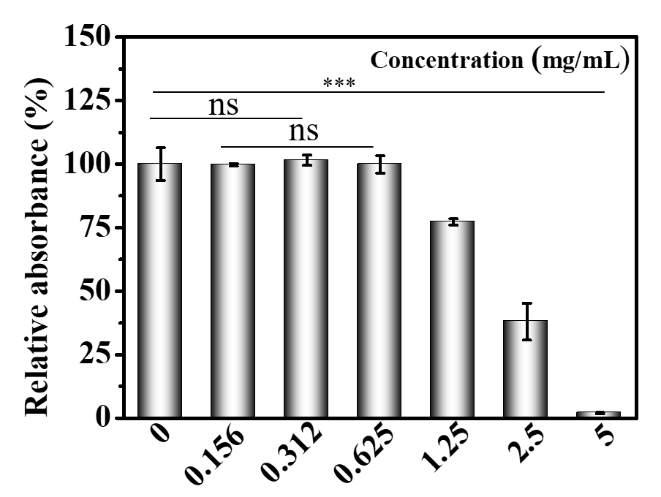


**Figure S4**. The antibacterial activity of NB toward MRSA (Data are presented as mean ± SD. ns: no significance difference, n=3, **P* < 0.05, ***p* < 0.01, and ****p* < 0.001).

**Figure S5**. The antibacterial activity of CAT-Ce6 toward MRSA in the dark environment (Data are presented as mean ± SD, n=3).

**Table S1**. Summary of sequence quality control

| **SampleID** | **Total_Reads** | **Clean_Rate%** | **Q20%** | **Q30%** |
| --- | --- | --- | --- | --- |
| Control | 31887214 | 97.84 | 98.91 | 96.26 |
| NB | 45038762 | 97.92 | 98.79 | 95.89 |


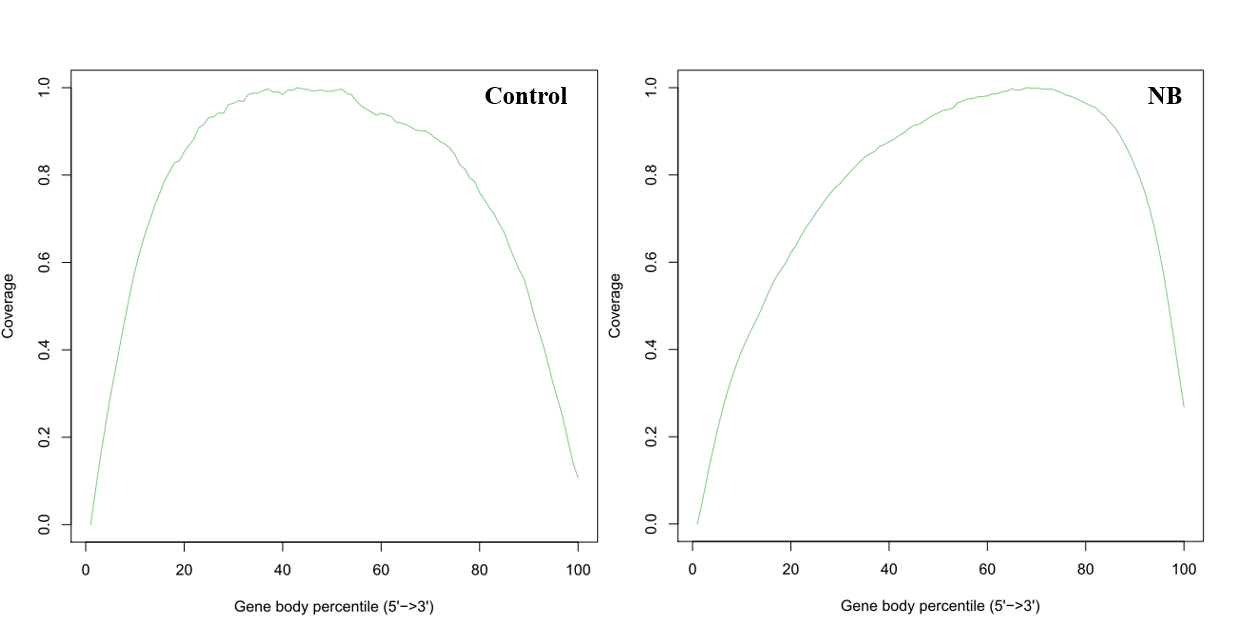


**Figure S6**. Gene coverage analysis of MRSA cells in different groups.


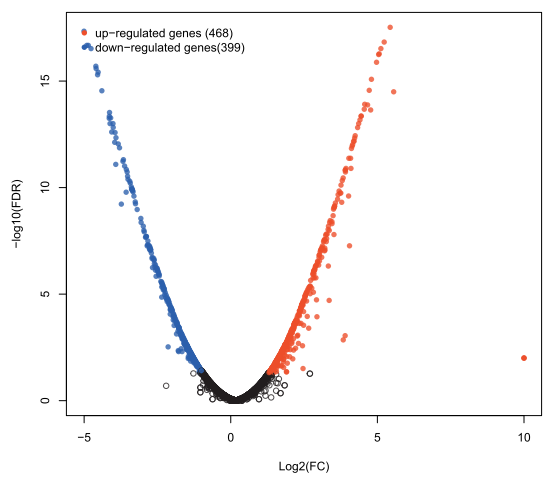


**Figure S7**. Volcano map for the distribution of differentially expressed genes between the control group and NB-treated group. Red dots indicate that the genes are significantly up-regulated, while blue dots are significantly down-regulated


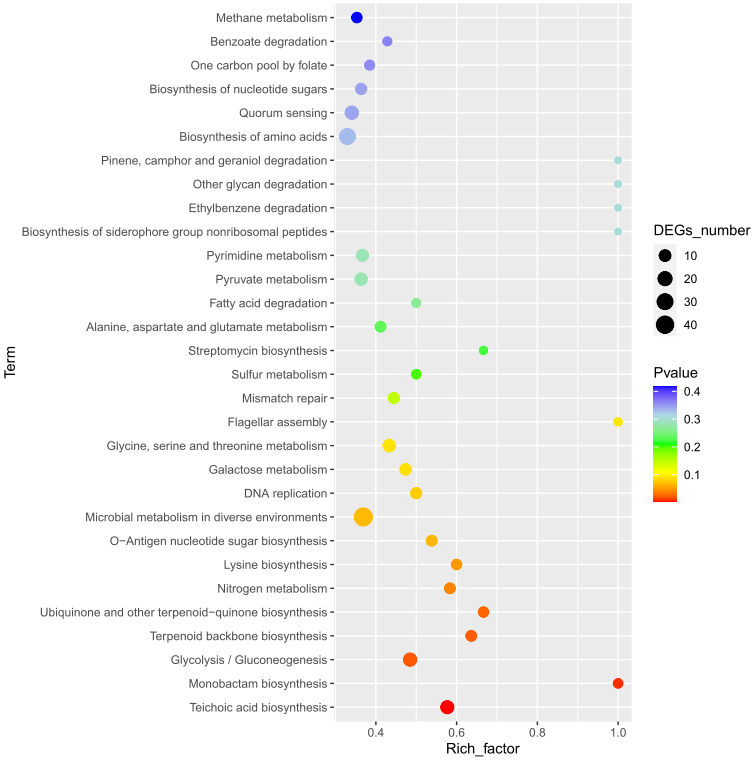


**Figure S8**. The KEGG enrichment analysis of DEGs in NB-treated MRSA.


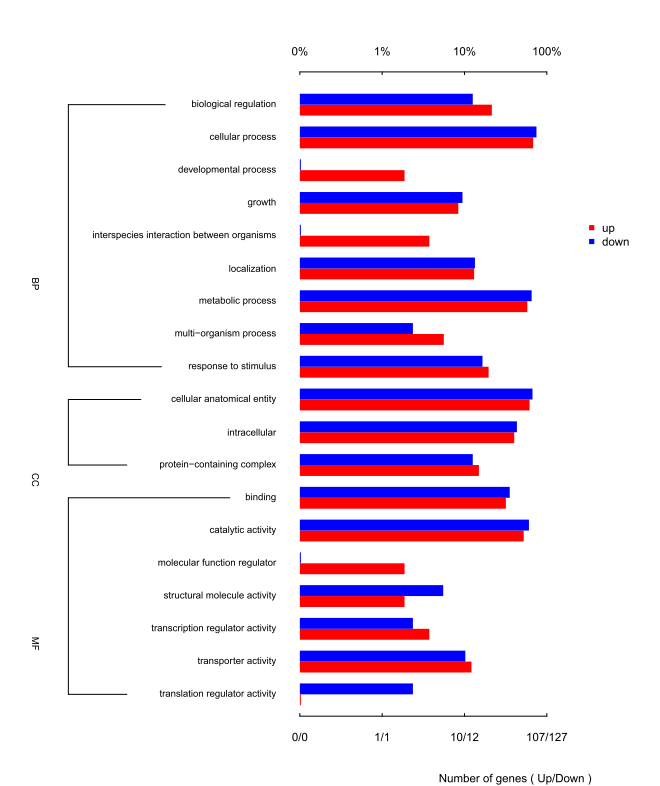


**Figure S9**. Scatterplot of GO enrichment analysis for Control versus NB.

**Table S2**. The primers used in this study

| **Gene** | **Primer Direction** | **Sequence (5'-3')** |
| --- | --- | --- |
| *ScdA* | Forward | GCAAGGTGAGGTAGTAGAC |
|  | Reverse | GCTTCTATCGGAGGTTCAT |
| *Asp23* | Forward | ACAAGCATACGACAATCA |
|  | Reverse | TTCAACAACTTCATCAGAGA |
| *CtsR* | Forward | AATGAAGATGTCGTTGAA |
|  | Reverse | TTAGTGATTCGGATGTAAC |
| 16S rRNA (Control) | Forward | GCGGTGGATGATGTGG |
|  | Reverse | GCTCGTTGCGGGACTTA |


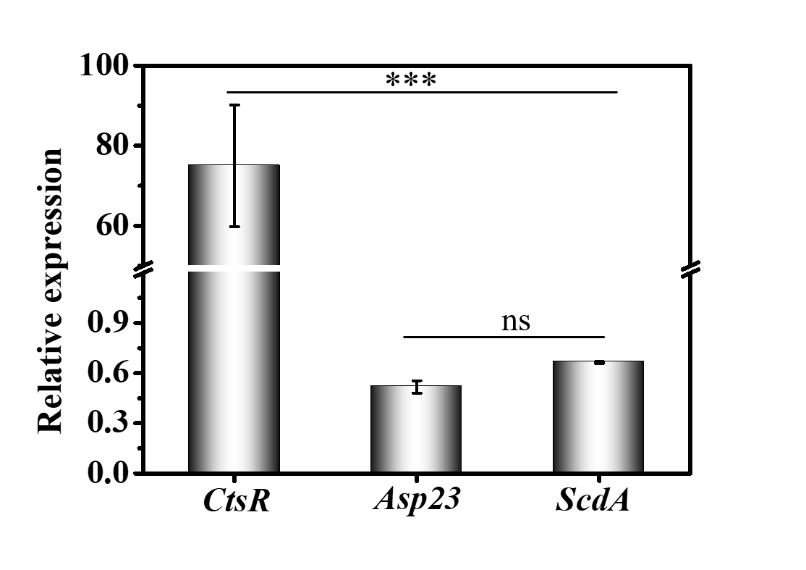


**Figure S10**. Relative expression levels of *CtsR*, *Asp23,* and *ScdA* after treatment with NB (Data are presented as mean ± SD. ns: no significance difference, n=3, *P < 0.05, **p < 0.01, and ***p < 0.001).

**Figure S11**. The bactericidal ability of gel(CaO_2_/alg/Glu/Gox) with different components against MRSA without 660 nm laser irradiation (Data are presented as mean ± SD).


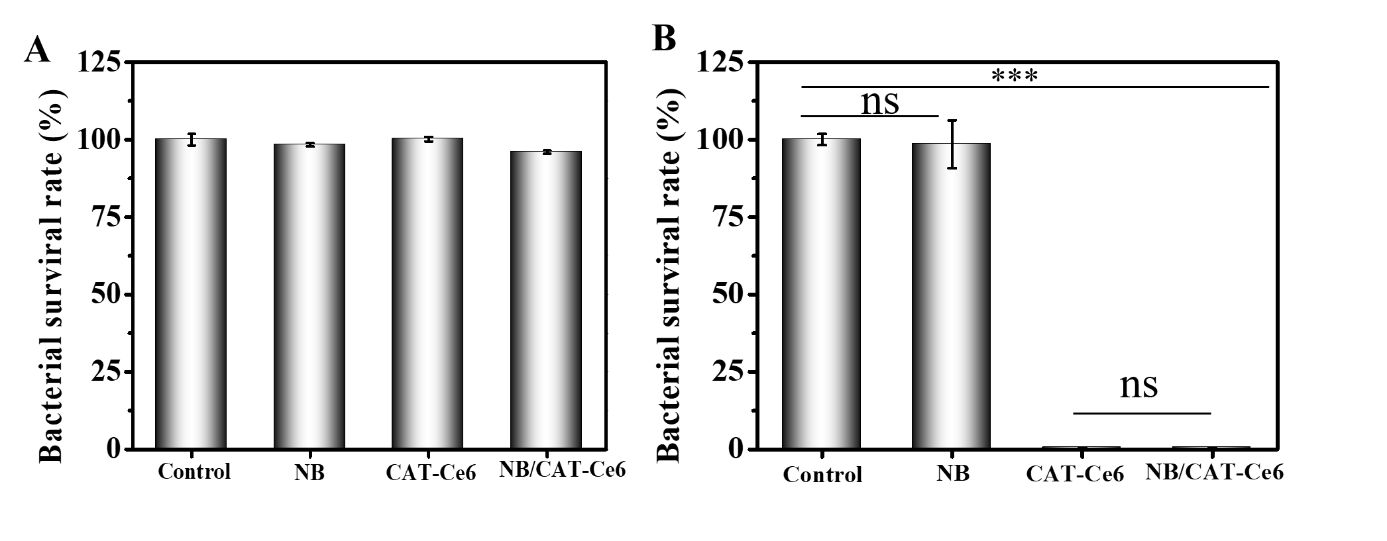


**Figure S12**. The bactericidal ability of gel(CaO_2_/alg/Glu/Gox) with different components against *E.coli* (A) without / (B) with 660 nm laser irradiation (Data are presented as mean ± SD. ns: no significance difference, n=3, **P* < 0.05, ***p* < 0.01, and ****p* < 0.001).


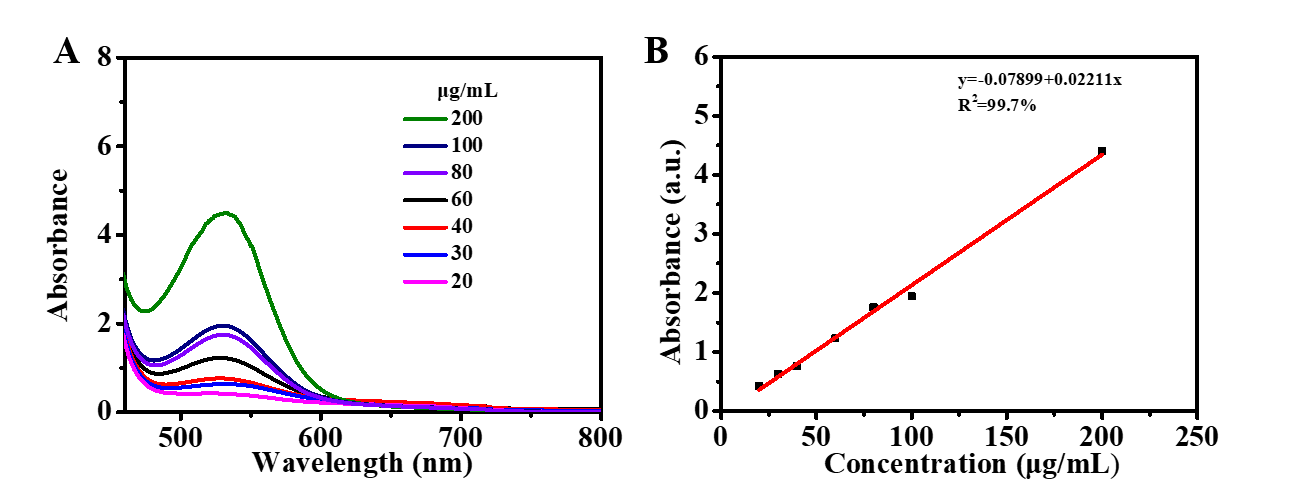


**Figure S13**. (A) Absorption spectra of different concentrations of NB and vanillin in the concentrated sulfuric acid solution. (B) The plot of absorbance at 530 nm versus the concentration of NB.

**Figure S14**. NB release profile from gel(CaO_2_/alg/Gox/Glu/NB/CAT-Ce6).


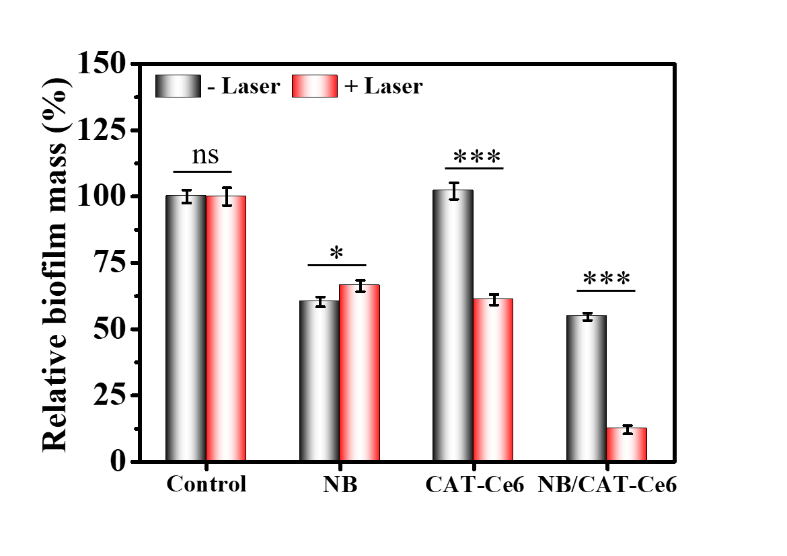


**Figure S15**. Evaluation of the antibiofilm activity of gel(CaO_2_/alg/Glu/Gox) with different compositions against *E. coli* (Data are presented as mean ± SD. ns: no significance difference, n=3, **P* < 0.05, ***p* < 0.01, and ****p* < 0.001)..


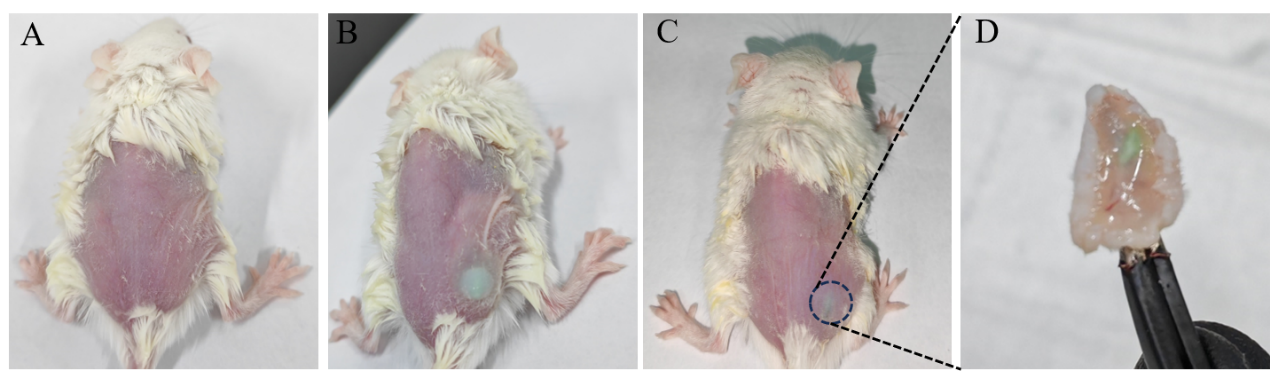


**Figure S16**. (A, B) Before and after subcutaneous injection of CaO_2_/alg/Gox/NB/CAT-Ce6 in diabetic mice. (C) After injection 2 hours. (D) Photos of the gel formed in the subcutaneous tissue.


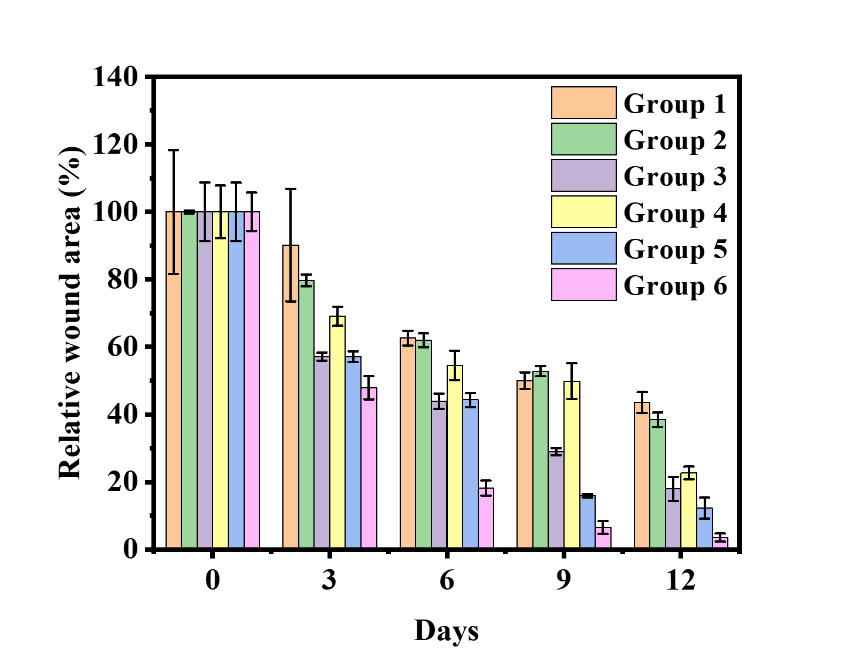


**Figure S17**. Changes in the abscess area over time. Group 1-6: no treatment (group 1), CaO_2_ + alg precursor solution (group 2), CaO_2_/alg/Gox precursor solution (group 3), CaO_2_/alg/CAT-Ce6 precursor solution (group 4), CaO_2_/alg/Gox/CAT-Ce6 precursor solution (group 5), and CaO_2_/alg/Gox/CAT-Ce6/NB precursor solution (group 6) (Data are presented as mean ± SD, n=3).


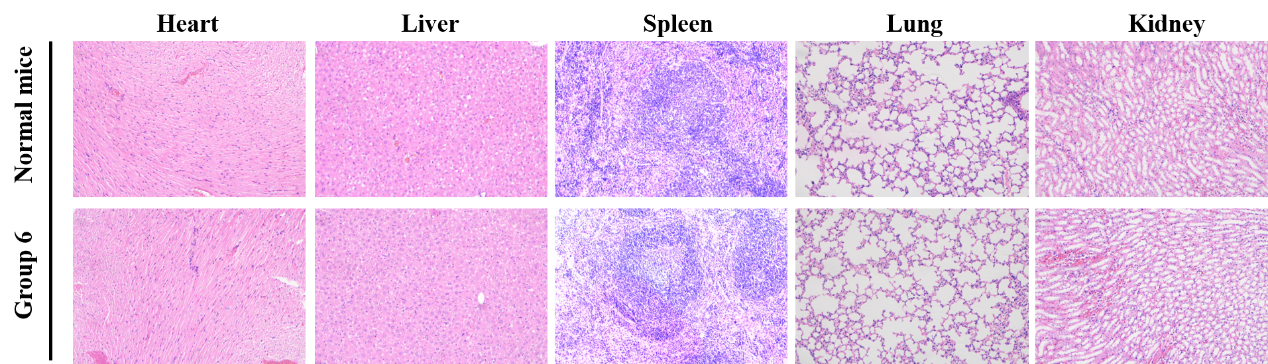


**Figure S18**. H&E staining of main organs in normal and gel(CaO_2_/alg/Gox/Glu/NB/CAT-Ce6)-treated mice. Magnification times = 200.
